# MicroRNA-181 Variants Regulate T Cell Phenotype in the Context of Autoimmune Neuroinflammation

**DOI:** 10.3389/fimmu.2017.00758

**Published:** 2017-07-19

**Authors:** Samira Ghorbani, Farideh Talebi, Wing Fuk Chan, Farimah Masoumi, Mohammed Vojgani, Christopher Power, Farshid Noorbakhsh

**Affiliations:** ^1^Department of Immunology, School of Medicine, Tehran University of Medical Sciences, Tehran, Iran; ^2^Shefa Neuroscience Research Center, Khatam Al-Anbia Hospital, Tehran, Iran; ^3^Department of Medicine (Neurology), University of Alberta, Edmonton, AB, Canada; ^4^Multiple Sclerosis Centre, University of Alberta, Edmonton, AB, Canada

**Keywords:** experimental autoimmune encephalomyelitis, multiple sclerosis, microRNA, miR-181, suppressor of mothers against decapentaplegic 7

## Abstract

**Background:**

Recent studies have revealed that multiple sclerosis (MS) lesions have distinct microRNA (miRNA) expression profiles. miR-181 family members show altered expression in MS tissues although their participation in MS pathogenesis remains uncertain. Herein, we investigated the involvement of miR-181a and miR-181b in the pathogenesis of MS and its animal model, experimental autoimmune encephalomyelitis (EAE).

**Methods:**

miR-181a and -b levels were measured in the central nervous system (CNS) of patients with MS and mice with EAE as well as relevant leukocyte cultures by real-time RT-PCR. To examine the role of the miRNAs in leukocyte differentiation and function, miR-181a and -b mimic sequences were transfected into cultured primary macrophages and purified CD4^+^ T cells which were then analyzed by RT-PCR and flow cytometry. Luciferase reporter assays were performed to investigate the interaction of miR-181a and -b with the 3′-UTR of potential target transcripts, and the expression of target genes was measured in the CNS of EAE mice, activated lymphocytes, and macrophages.

**Results:**

Expression analyses revealed a significant decrease in miR-181a and -b levels in brain white matter from MS patients as well as in spinal cords of EAE mice during the acute and chronic phases of disease. Suppression of miR-181a was observed following antigen-specific or polyclonal activation of lymphocytes as well as in macrophages following LPS treatment. Overexpression of miR-181a and -b mimic sequences reduced proinflammatory gene expression in macrophages and polarization toward M1 phenotype. miR-181a and -b mimic sequences inhibited Th1 generation in CD4^+^ T cells and miR-181a mimic sequences also promoted Treg differentiation. Luciferase assays revealed Suppressor of mothers against decapentaplegic 7 (Smad7), as a direct target of miR-181a and -b.

**Conclusion:**

Our data highlight the anti-inflammatory actions of miR-181a and -b in the context of autoimmune neuroinflammation. miR-181a and -b influence differentiation of T helper cell and activation of macrophages, providing potential therapeutic options for controlling inflammation in MS.

## Introduction

Multiple sclerosis (MS) is a chronic and progressive inflammatory neurological disorder that is defined by central nervous system (CNS) infiltration of autoreactive lymphocytes followed by demyelination and axonal injury (1). Evidence points to the involvement of both innate and adaptive immune mechanisms in disease process (2). It is widely believed that activation of neuroantigen-reactive T cells occurs in the peripheral immune system in the beginning of disease; activated T cells then enter the CNS where they are reactivated and cause local microglial activation, monocyte infiltration, and oligodendrocyte and myelin damage. To gain insight into molecular changes that occur in the CNS during disease, many studies have focused on transcript and protein expression levels within and around demyelinating lesions in MS (3–5). These studies have shown altered expression of inflammatory as well as structural genes in the CNS of MS patients and have provided important information about the pathogenesis and potential therapeutic targets for disease (3).

MicroRNAs (miRNAs) are non-coding RNA molecules that regulate gene expression through sequence-specific binding to target mRNAs, leading to translational silencing or transcript degradation (6). Experimental evidence indicates that miRNAs are involved in the differentiation and function of immune cells and play important roles in immune response (7–10). miRNA dysregulation has been reported in numerous human diseases, including cancer, infectious diseases, and autoimmune/autoinflammatory disorders (e.g., MS), the focus of this paper (11–14). miRNA profiling studies in blood and CNS from MS patients have revealed perturbed expression of multiple miRNAs in blood cells and the CNS tissue (13). Some of the dyregulated miRNAs have been shown to be associated with pathogenically relevant biological processes, including cell death and apoptosis, neuroprotection, and leukocyte activation and function (15–17). Members of the miR-181 family are among dysregulated miRNAs in the CNS of patients affected by MS (13, 14). The miR-181 family is highly conserved and consists of four members (miR-181a, miR-181b, miR-181c, and miR-181d) in both humans and mice. miR-181a and miR-181b are highly expressed in the brain, bone marrow, spleen, and thymus (10, 18). Prior studies have reported on the roles of miR-181 family members in development and function of immune cells, including their role in B cell and T cell differentiation and activities (10, 19). Moreover, gene ontology analysis of miR-181a and -b’s predicted targets has shown an overrepresentation of immune pathways including T-cell receptor signaling and transforming growth factor (TGF)-β signaling (20). In the context of MS, both up- and downregulation of miR-181 mature isoforms have been reported in MS brains, this is likely a consequence of the degree of inflammation and tissue location with respect to MS lesions (13, 14). Nonetheless, there is limited information regarding the role of miR-181a and -b in MS pathogenesis. In this study, we investigated the potential impact of miR-181a and -b on the pathogenesis of MS, using the established model for MS, experimental autoimmune encephalomyelitis (EAE) as well as *in vitro* culture systems and human brain tissues. miR-181a and -b expression levels together with their actions were then analyzed in macrophage and T cell differentiation assays. Targets of miR-181a and -b with known roles in immune pathways were also identified in relevant databases and interaction of miRNAs with 3′-UTR region of targets were examined using molecular assays.

## Materials and Methods

### Human Brain Tissue Samples

The use of autopsied brain tissues were approved under the protocol number 2291 by the University of Alberta Human Research Ethics Board (Biomedical), and written informed consent documents were signed for all samples collected from age- and sex-matched subjects (10 non-MS patients and 10 patients with MS), and samples were stored at −80°C (14, 21). MS patients included eight cases of secondary progressive MS and two cases of primary progressive MS. All MS patients had advanced disease (EDSS 7.0–9.5 at the time of death). Non-MS cases included seven cases of non-neurological disease (cancer, sepsis, and myocardial infarction), two cases of ALS, and one stroke case. The interval between death to autopsy ranged from 12 to 36 h. In each MS patient, LFB and H&E staining were performed on multiple brain sections, and tissue samples were collected from normal appearing white matter (NAWM) juxtaposed to the lesions. Details of MS patients and non-MS controls are shown in Table S1 in Supplementary Material.

### Experimental Autoimmune Encephalomyelitis Induction

C57BL/6 wild-type mice (6 weeks old) were purchased from the Pasteur Institute of Iran and maintained in the animal facility of Tehran University of Medical Sciences. At 12 weeks of age, mice (*n* = 30) were injected subcutaneously with MOG 35-55 peptide emulsified in complete Freund’s adjuvant (CFA) at two sites. The animals also received two intraperitoneal injections of pertussis toxin administered on the day of immunization and 24 h later, as instructed by the manufacturer (EK-2110, Hooke Kit™ MOG 35-55/CFA Emulsion PTX). Clinical assessment of EAE was performed daily for 30 days postimmunization using a 0- to 15-point scoring scale (22). All experiments were performed in accordance with guidelines from Animal Care Committee of Tehran University of Medical Sciences. Spleen and CNS tissues were dissected from EAE mice at three time points following disease induction including: pre-onset (before the appearance of symptoms, approximately Day 10 postimmunization), acute phase (at the peak of the disease), and a late phase here called chronic phase (Days 24–30 postimmunization). Spinal cord tissue samples from EAE and control mice were stored at −80°C.

### RNA Isolation, cDNA Synthesis, and Real-time PCR

Total RNA was isolated from tissue samples and cells using miRNeasy Mini Kit (Qiagen) and stored at −80°C. First-strand cDNA synthesis was performed with 0.5–1 µg total RNA using miScript II RT Kit (Qiagen) for miRNA analyses and TAKARA kit for mRNA expression analyses according to the manufacturer’s instructions (TAKARA). Real-time RT-PCR was performed on a Bio-Rad machine using Syber Green method (Primer sequences are shown in Table S2 in Supplementary Material). For miRNA detection, cDNA was amplified using miScript primers (Qiagen). miRNA expression data were normalized against snord68 and snord72 transcript levels (Qiagen) while β-actin and GAPDH genes were used to normalize mRNA expression.

### Cell Cultures and MOG Stimulation

To perform *in vitro* MOG stimulation experiments, splenocyte cultures were prepared from MOG-immunized mice. Animals were sacrificed 10 days after immunization, and spleens were removed under sterile conditions. Spleen tissues were homogenized and splenocytes were isolated using Ficoll-Hypaque density gradient centrifugation. 2 × 10^6^ cells were cultured in the presence of different concentrations of MOG35-55 (MOG in TC Media, 100×, Hooke labs) in RPMI 1640 medium (Gibco) supplemented with 5% FBS (Gibco). Cells were harvested after 12, 24, and 72 h of incubation. In parallel experiments, splenocytes prepared from 6- to 8-week-old C57BL/6 mice were stimulated with anti-CD3 (0.5 µg/ml) and anti-CD28 (0.2 µg/ml) antibodies (eBioscience) for different time points. For differentiation experiments, naive CD4^+^ T cells were purified using mouse naïve CD4^+^ T Cell Isolation Kit II (Miltenyi Biotec), through depletion of non-CD4^+^ T cells, i.e., CD8a, CD11b, CD11c, CD19, CD25, CD45R (B220), CD49b (DX5), CD105, MHC Class II, Ter-119, and TCRγ/δ immunopositive cells. Bone marrow-derived macrophages (BMDMs) were prepared from femurs and tibiae of C57/BL6 mice, as previously described (23, 24). Cells were differentiated in RPMI 1640 culture medium containing 10% FBS, 100 U/ml penicillin, 100 mg/ml streptomycin, and 50 ng/ml recombinant M-CSF (eBioscience). Cells were seeded in 24-well plates at a density of 1 × 10^6^ cells and treated with LPS (10 and 100 ng/ml) after 6 days for 12 h.

### miRNA Transfections

Transfection assays were performed using Hiperfect Transfection Reagent according to manufacturer’s instructions (Qiagen). miRNA-181a and -b mimic as well as scrambled sequences were purchased from Qiagen (Syn-mmu-miR-181a miScript miRNA Mimic, Syn-mmu-miR-181b miScript miRNA Mimic, AllStars Negative Control siRNA). Briefly, 3 µl of Hiperfect Transfection Reagent was added to 100 µl of serum-free DMEM medium containing miR-181a or miR-181b mimics or negative control at a final concentration of 50 nM. The volume of cell culture medium in 24-well plates was adjusted to 600 µl with medium containing 10% FBS. To evaluate the transfection efficiency, we monitored green fluorescent protein (GFP) expression using fluorescence microscope. The percentage of GFP-positive cells varied between 60 and 65% for lymphocytes and 80 and 85% for HEK cells. We also measured miR181a and -b expression in transfected lymphocytes by real-time PCR (Figure S1 in Supplementary Material).

### CD4^+^ T Cell Activation and Polarization

Four hours after transfection with miRNA sequences, naïve CD4^+^ T cells were transferred to anti-CD3 coated wells (1 µg/ml) and were treated with soluble anti-CD28 (0.5 µg/ml) antibodies (eBioscience). To polarize cells toward T regulatory cells, IL2 (20 ng/ml), TGF-β (50 ng/ml), anti-IFN-γ (10 mg/ml), and anti-IL-4 (10 mg/ml) were added to cells. To polarize cells toward a Th_17_ phenotype, TGF-β (5 ng/ml), IL-6 (100 ng/ml), anti-IFN-γ (10 mg/ml), anti-IL-4 (10 mg/ml), and IL23 (50 ng/ml) were applied to cultures for 96 h. For polarization toward a Th1 phenotype, IL2 (20 ng/ml), IL-12 (50 ng/ml), and anti-IL-4 (10 mg/ml) were applied for 96 h. All antibodies and cytokines were purchased from Biolegend.

### Flow Cytometry Analysis

Following T cell transfection and treatment, cells were evaluated for the expression of Th1, Treg, and Th17 cells differentiation markers using flow cytometry. Cells were first exposed for 6 h to a cell activation cocktail containing phorbol-12-myristate 13-acetate, ionomycin, and Brefeldin A (Biolegend, 423303). Cells were then immunolabeled with PerCP-labeled anti-mouse CD4 (Biolegend, 100537) and APC-labeled anti-mouse CD3 antibodies (Biolegend, 100235) for 30 min (4°C). Cells were then fixed with 1 ml/tube BioLegend’s fixation buffer, at room temperature in the dark for 20 min. Cells were permeabilized with 1 ml BioLegend’s permeabilization buffer (Biolegend, 422601). Permeabilized cells were incubated with PE-labeled anti-mouse IL17A (Biolegend, 506903), PE-labeled anti-mouse IFN-γ (Biolegend, 505807) or PE-labeled anti-mouse Foxp3 antibodies (Biolegend, 126403) in the dark for 20 min. Finally, immunolabeled cells were analyzed by a FACS Calibur Flow Cytometer (BD Biosciences).

### Luciferase Reporter Assays

Luciferase reporter assays were used to verify direct interactions between the miRNAs and the target genes. 3′-UTR region of *Smad7*, suppressor of cytokine signaling-3 *(Socs*3), and *Tgfbr*1 were PCR-amplified from mouse genomic DNA, using primers containing appropriate restriction sites and then cloned into psiCheck vector, a Renilla luciferase reporter vector (Promega). The following primer sequences were used to amplify specific 3′-UTR fragments.

Smad7-F 5′GGTGGT**C″TCGAG**AGGCCACCGTTCAAACTACT3′,
Smad7-R 5′ATAT**GC″GGCCGC**TCCTTTCCTCTCTCAAAGCACT3′
Socs3-F 5′GGTGGT**C″TCGAG**AAAAATCCAGCCCCAACGT3′
Socs3-R 5′ATAT**GC″GGCCGC**TTTCTCCCCAACACAGGACC3′,
Tgfbr1-F 5′GCAGCA**C″TCGAG**GCTGTTGTTCTGTTATAGCCC3′,
Tgfbr1-R 5′CTAA**GC″GGCCGC**GGTGGGAAAAAGTTGTTTATTTGC3′

Correct orientation and sequences were confirmed by restriction digestion and sequencing.

To assess the impact of the miRNAs, recombinant plasmid DNA (200 ng) and miRNA mimics (100 nM) were co-transfected into HEK293T cells using Attractene Transfection Reagent (Qiagen). Co-transfection with a scrambled miRNA sequence was used as a control. miR-181a and miR-181b mimics, negative control, and Attractene transfection reagent were purchased from Qiagen (Syn-mmu-miR-181a miScript miRNA Mimic, Syn-mmu-miR-181b miScript miRNA Mimic, AllStars Negative Control siRNA). Luciferase activity was measured using the Dual Luciferase system (Promega) 48 h post-transfection. Renilla luciferase levels were normalized to firefly luciferase activity as the internal transfection control.

### Statistical Analysis

Statistical analyses were performed using SPSS, and graphs were prepared using GraphPad Prism. Statistical significance was determined by performing ANOVA followed by appropriate *post hoc* testing for multiple comparisons and Student’s *t*-test or Mann-Whitney U test for two-group comparisons. *p* values below 0.05 were considered statistically significant. All values are shown as average ± SEM.

## Results

### miR-181a and miR-181b Are Downregulated in the CNS of MS Patients and EAE Animal Model

**Figure 1:**
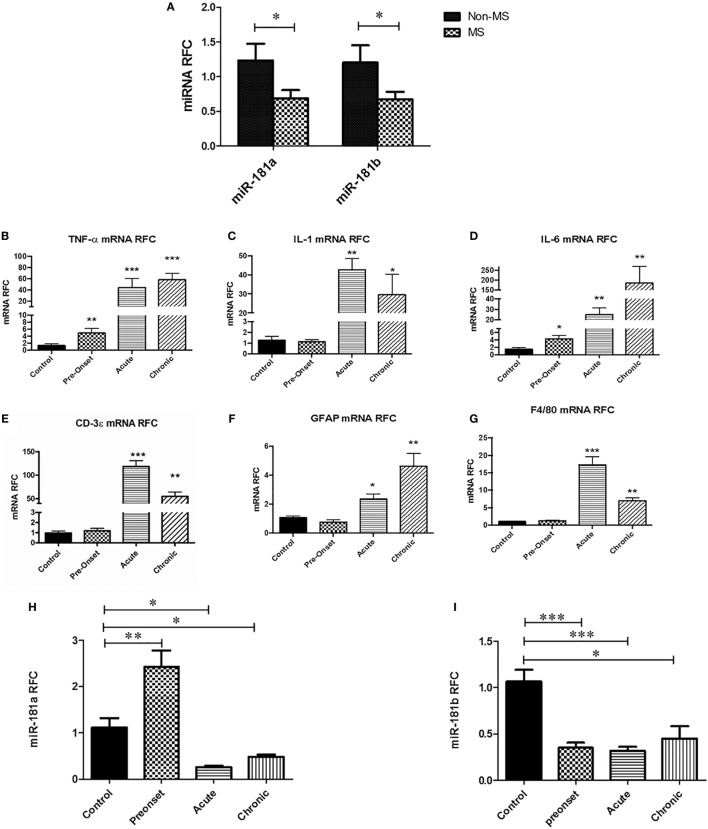
miR-181a and miR-181b expression levels are decreased in the central nervous system of multiple sclerosis (MS) patients and experimental autoimmune encephalomyelitis (EAE) mice. Expression analysis on brain autopsy samples shows levels of miR181a and miR-181b in the brains of MS patients (*n* = 10) compared with non-MS controls (*n* = 10) (Mann–Whitney *U* test, *p ≤ 0.05) **(A)**. mRNA expression analyses on lumbar spinal cords from EAE mice show levels of inflammatory cytokines Tnfa **(B)**, Il1 **(C)**, and Il6 **(D)** during disease. Expression of T cell markers Cd3e **(E)**, astrocyte marker Gfap **(F)** and monocytoid cell marker F4/80 **(G)** were also measured at different phases of disease. Expression of miR-181a **(H)** and miR-181b **(I)** were quantified in the spinal cord tissue derived from EAE mice at different stages of disease. Data are shown as mean ± SEM (*n* = 10, ANOVA-Tukey *post hoc*; *p < 0.05, **p < 0.01, ***p < 0.001, relative to control).

To examine the expression of miR-181a and -b in the CNS during MS disease, we first performed a quantitative expression analysis on white matter samples obtained from autopsied brain tissues of MS patients and non-MS controls. In MS cases, samples were taken from white matter tissue juxtaposed to the lesions. Real-time RT-PCR showed significant reduction in miR-181a and miR-181b levels in MS brain samples compared with non-MS controls (Figure 1A). Given the reduced expression in MS tissues, we next examined miRNA expression levels in the CNS tissues from mice with EAE at three time points after disease induction; before the onset of neurobehavioral signs, at the acute and the chronic disease phases. Expression analyses of inflammation-related genes showed upregulation of *Tnfa* and *Il6*, but not *Il1b*, in EAE lumbar spinal cords at the pre-onset phase of disease compared with control animals’ spinal cords (Figures 1B–D). As expected, all three transcripts showed substantial induction at acute and chronic phases of disease. Expression of *Cd3e* (lymphocyte marker), *Gfap (*astrocyte marker) and *F4/80* (monocytoid cell marker) also showed significant induction at acute and chronic phases (Figures 1E–G). Interestingly, analysis of the expression of murine miR-181a and -b in lumbar cord tissue samples from EAE mice in three phases of disease (pre-onset, acute and chronic) showed significant reduction for both miR-181a and -b levels in acute and chronic phases (Figures 1H,I). At the pre-onset phase, miR-181a showed an increase while miR-181b was reduced similar to the acute and chronic phases.

### miR-181a and -b Expression Are Reduced following Immune Cell Activation

**Figure 2:**
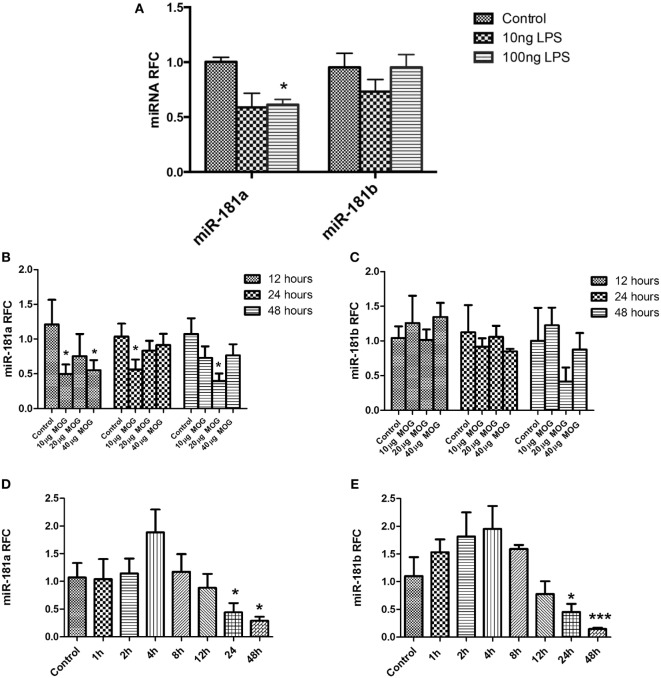
miR-181a and miR-181b expression is decreased in activated leukocytes. Primary macrophage cultures were treated with LPS (10 and 100 ng/ml) for 12 h and the expression of miR-181a and miR-181b were measured **(A)**. Splenocytes prepared from MOG- immunized mice were stimulated *in vitro* with different concentrations (10, 20, and 40 µg/ml) of MOG peptide. Expression of mir-181a **(B)** and miR-181b **(C)** were determined by quantitative real-time PCR analysis at three time points. In separate experiments, splenocytes were stimulated with anti-CD3 and anti-CD28 for indicated time points and expression of miR-181a **(D)** and miR-181b **(E)** were quantified. Data are shown as mean ± SEM (*n* = 3, Experiment was repeated twice. ANOVA-Tukey *post hoc*; *p < 0.05, ***p < 0.001, relative to control).

Different types of immune cells including T cells and macrophages are involved in MS/EAE neuroinflammation and pathogenesis. To examine whether diminished expression of miR-181 isoforms might occur in these cells following activation, we evaluated miR-181a and -b expression levels in primary macrophages and lymphocytes following cell activation. To investigate expression levels in primary macrophages we established BMDM cultures from C57/BL6 mice. Activation of macrophages during autoimmune neuroinflammation occurs in response to complex alterations in CNS microenvironment, where multiple cytokines, chemokines, and other inflammatory mediators are present. Such an environment is difficult to recreate *in vitro*; hence we used the simplified model of LPS stimulation of BMDMs, a model which has been widely used to simulate some aspects of monocytoid cell activation (25, 26). Mouse BMDM cells were stimulated with two different concentrations of LPS (10 and 100 ng/ml) for 12 h. Evaluation of transcript levels revealed significant reduction of miR-181a levels after treatment with 100 ng/ml LPS concentration. Nonetheless, mir-181b levels did not show any significant changes following LPS stimulation (Figure 2A). We next asked whether activation of T cells might influence the expression of miR-181a and -b in these cells. To this end, both antigen-specific stimulation and polyclonal activation of T cells were performed. For antigen-specific stimulation, splenocytes derived from MOG-immunized animals were treated with three different concentrations of MOG peptide (10, 20, and 40 µg/ml) for three different time points. Expression analysis of MOG-stimulated cells showed significant reduction of miR-181a levels at 10 and 40 µg/ml concentrations of MOG at the 12-h time point (Figure 2B). miR-181a reduction was also observed at 10 µg/ml MOG concentration at 24-h time point (Figure 2B). However, miR-181b levels did not show statistically significant changes after MOG stimulation (Figure 2C). To examine the expression levels of miR-181 isoforms in T cells following polyclonal activation, we stimulated splenocytes with anti-CD3/CD28 antibodies and studied the transcript levels at several time points following stimulation. To confirm activation of T cells following anti-CD3/CD28 treatment we measured both the proliferation of CD3 positive cells by CFSE staining and also their IL2 and interferon gamma expression by real-time PCR (Figure S2 in Supplementary Material). As shown in Figures 2D,E, activated T cells showed reduced levels of miR-181a and miR-181b at 24 and 48 h time points after stimulation. Overall, these data indicated that activation of immune cells could be associated with diminished expression of miR-181 isoforms, which in turn might play a role in subsequent pathogenic events.

### miR-181a and -b Regulate Activation and Polarization of Macrophages

**Figure 3:**
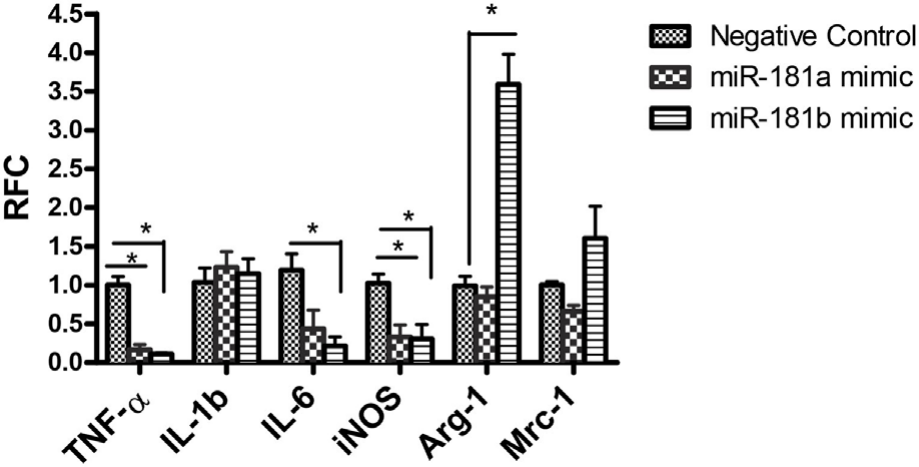
miR-181a and miR-181b modulate macrophage activation and polarization. Primary macrophages were transfected with miR-181a, miR-181b, or negative control sequences and then exposed to LPS (100 ng) for 12 h. The expression of inflammatory cytokines Tnfa, Il1, and Il6 together with M1/M2 markers iNOS, Mrc1, and arginase were then analyzed. Data are shown as mean ± SEM (*n* = 3, experiment was repeated twice. ANOVA-Tukey *post hoc*; *p < 0.05).

To investigate the functional significance of miR-181 isoforms in proinflammatory responses of macrophages, BMDM cells were transfected with miR-181a or -b mimics or a negative control sequence, prior to LPS stimulation. Expression analysis for putative inflammatory cytokines *Tnfa, Il1b*, and *Il6* showed diminished levels of *Tnfa* and *Il6* transcripts in cells transfected with miR-181a or miR-181b sequences (Figure 3), whereas *Il1b* levels were unaffected. Asking whether miR-181 overexpression might affect the differentiation of macrophages toward M1 or M2 phenotypes, we examined the expression of iNos, a key M1 marker, and arginase and Mrc1 as M2 markers in transfected cells. iNos expression was markedly reduced in miR-181a or -b overexpressing cells. For M2 markers, *arginase* was induced in miR-181b overexpressing macrophages (Figure 3). Since LPS drives an M1 phenotype, increased levels of M2 markers in miRNA-transfected LPS-stimulated cells is likely reflective of decreased M1 differentiation. We also analyzed the expression of M1/M2 markers in resting macrophages (without LPS stimulation). Overexpression of miR-181b in resting macrophages resulted in decreased levels of Il6 together with a mild increase in arginase (M2 associated gene) but the increase did not reach statistical significance (Figure S3 in Supplementary Material). Altogether, the data indicate the miR-181a and -b are likely to act as negative regulators of macrophage activation and they might also tip the balance of macrophage differentiation away from the proinflammatory M1 phenotype.

### miR-181 Family Members Regulate the Differentiation of Th1 Cells and Tregs

**Figure 4:**
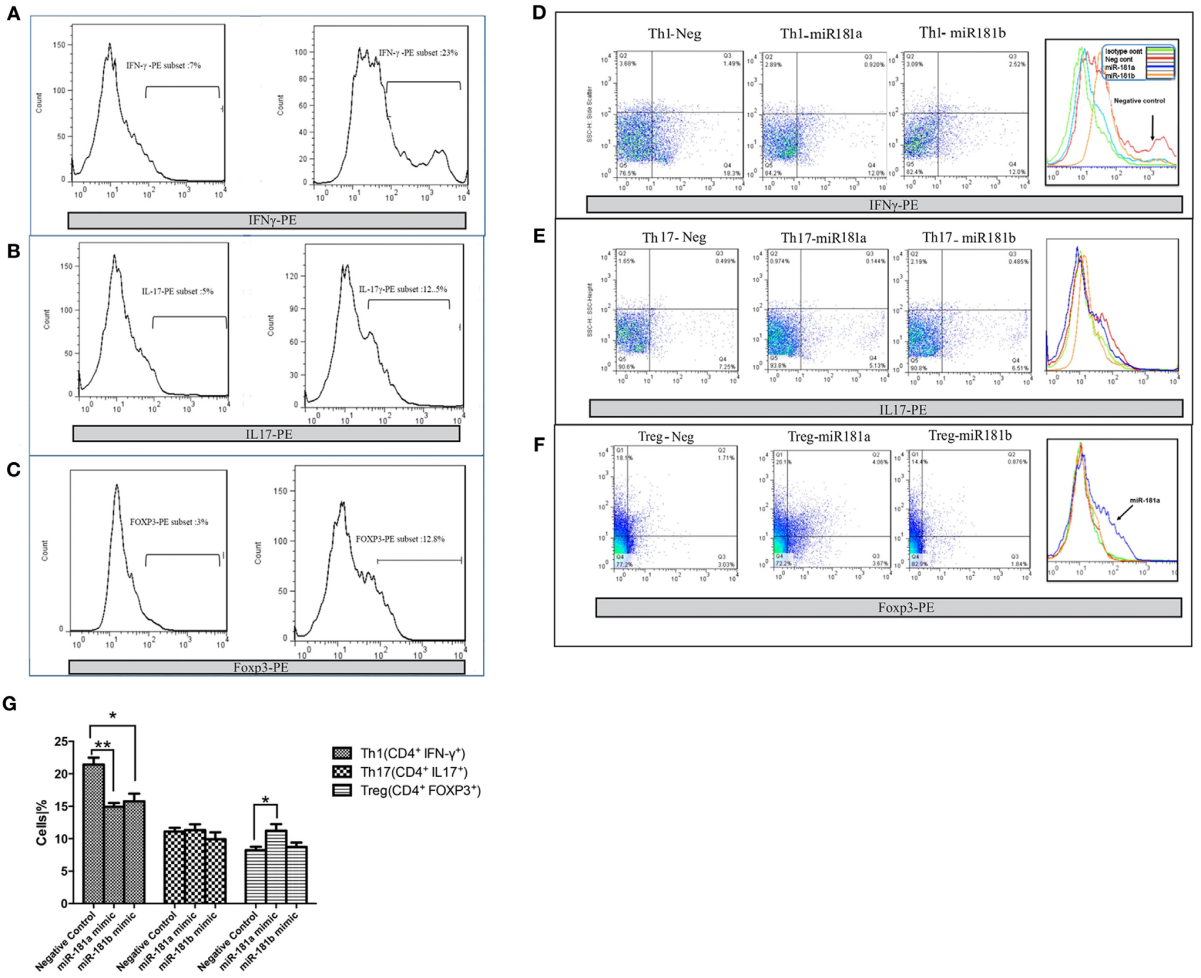
miR-181a and miR-181b regulate CD4^+^ T cells differentiation. miR-181a, miR-181b, and negative control sequences were transfected into purified naïve CD4^+^ T cells, which were then activated and polarized. The frequencies of Th1 **(A)**, Th17 **(B)**, and Treg cells **(C)** were determined by flow cytometry 4 days later. Representative histograms show the frequency of IFNγ immunopositive Th1 cells **(D)**, IL17 immunopositive Th17 **(E)**, and FoxP3 immunopositive Treg cells **(F)**. Quantification of cell frequencies is shown in the bar graph **(G)**. Percentages of positive cells in CD4^+^ T cells are presented as mean ± SEM (*n* = 3). Data are from a single experiment representative of three independent experiments (ANOVA-Tukey *post hoc*; *p < 0.05, **p < 0.01).

To evaluate the potential role of miR-181 isoforms in differentiation of T helper cells, CD4^+^ T cells were purified from mouse splenocytes. Purified naïve CD4^+^ T cells were transfected with miR-181a and -b mimic sequences and cultured in Th1, Th17, or T regulatory polarizing conditions for a period of 4 days, as described in Section “Materials and Methods.” Using flow cytometry, the frequencies of Th1, Th17, and Treg cells in CD4^+^ T cells were determined after polarization and activation. Exposure of T cells to Th1 polarizing conditions, enhanced the frequency of IFN-γ immunopositive cells compared with undifferentiated (Th0) cells, as expected (Figure 4A). Interestingly, overexpression of miR-181a and miR-181b mimic sequence reduced the frequency of Th1 cells (Figures 4D,G). Likewise, Th17 polarizing conditions increased IL17 immunopositive cells (Figure 4B), but the frequency of these cells did not reveal any difference following miR-181a or miR-181b transfection (Figures 4E,G). T cells exposed to T regulatory polarizing conditions revealed enhanced frequency of FoxP3 immunoreactive cells (Figure 4C). Interestingly, miR-181a overexpression led to a significant increase in the frequency of these induced Treg cells. The effect on Treg differentiation was negligible for miR-181b (Figures 4F,G). Overall, these data suggested that miR-181a and miR-181b diminished polarization of activated T cells toward a Th1 phenotype, a finding that was associated with increased Treg differentiation for miR-181a isoform.

### miR-181a and -b Directly Interact with and Regulate Smad7 Signaling Molecule in Cells

**Figure 5:**
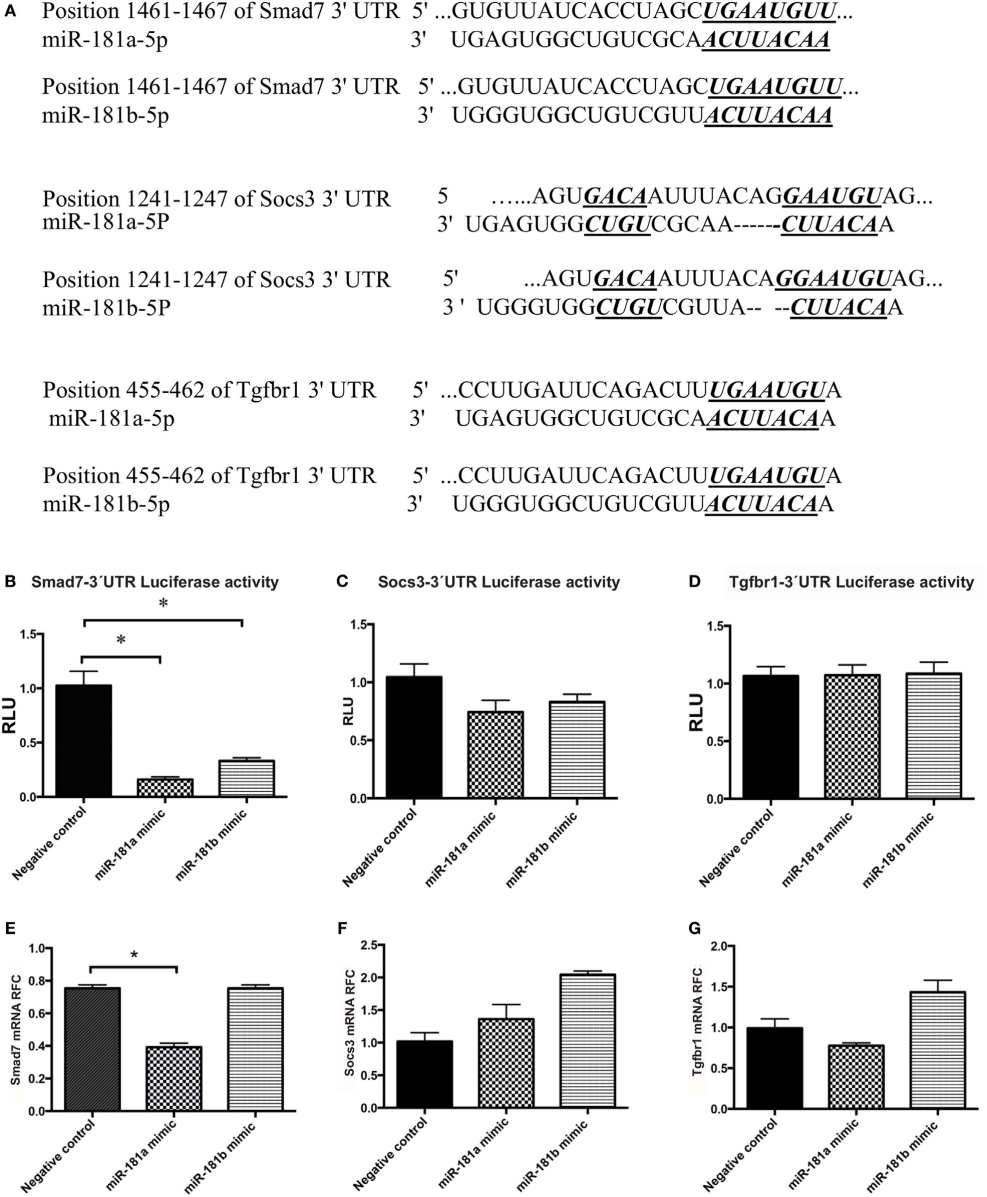
Smad7 transcripts are targeted by miR-181a and miR-181b. The sequence of the predicted binding site for miR-181a and miR-181b are shown on 3′-UTR of mouse Smad7, Socs3, or Tgfbr1 mRNA (in italic and bold) **(A)**. Luciferase activity was measured after co-transfection of 3′-UTR-containing vectors with microRNA sequences into HEK cells. Normalized luciferase activity levels are shown for Smad7 3′-UTR construct **(B)**, Socs3 3′-UTR construct **(C)**, and Tgfbr1 3′-UTR construct **(D)**. Renilla luciferase activities were normalized against internal Firefly luciferase. Levels of target transcripts were also quantified in miR-181a and -b transfected lymphocytes. Expression levels are shown for Smad7 **(E)**, Socs3 **(F)**, and Tgfbr1 **(G)**. Data are shown as means ± SEM. *n* = 5, experiment was repeated twice (ANOVA, Tukey *post hoc*, *p < 0.05).

MicroRNAs exert their effects through interactions with protein-coding transcripts. Target prediction algorithms like TargetScan can be used to obtain a potential list of mRNA targets for each miRNA sequence. Interestingly, gene ontology and pathway analysis performed on miR-181 predicted targets has shown an overrepresentation of TCR signaling and TGF-β signaling pathways among miR-181a and -b’s predicted targets (20). While prediction algorithms provide some insight about the potential mRNA targets of a particular miRNA, molecular analyses are required to verify miRNA/mRNA physical interactions inside the cells. Among miR-181a and -b predicted targets, we focused on molecules with known roles in T cell differentiation or cytokine signaling which also showed miRNA binding site conservation for miR-181a and -b in human and mouse (Figure 5A). Based on these factors, three genes were selected for experimental verification; *Smad7*, suppressor of cytokine signaling-3 (*Socs*3), and tumor growth factor β receptor 1(*Tgfbr1*). Smad7 is a negative regulator of Tgfbr1 signaling pathway and is known to drive Th1 response and suppress Treg cells (27). Socs3 is a key negative regulator of STAT signaling pathway, especially STAT3 (28, 29) and it can inhibit Th17 cells differentiation. Tgfbr1 is also known for mediating TGFb effects on different types of leukocytes, including its effects in promoting Treg differentiation.

A luciferase reporter assay system was used to examine the ability of the miR-181a and -b to knock down the expression of these target genes. The 3′-UTR region of the genes were PCR-amplified and cloned downstream of luciferase coding sequence into a psiCheck vector, as described in Section “Materials and Methods.” PsiCheck vectors containing the 3′-UTR of Smad7, Socs3, or Tgfbr1 mRNA were co-transfected along with miR-181a or miR-181b mimic sequences, or a scrambled negative control sequence into HEK293T cells. Renilla luciferase activity was then measured in the lysates of the cells and normalized against the background (firefly) luciferase activity. As shown in Figure 5B, cells transfected with a Smad7-3′-UTR-containing plasmid showed significant reduction in luciferase activity following transfection with miR-181a and -b, indicating the interaction between miRNA and the 3′-UTR region. Similar experiments with Socs3 and Tgfbr1 3′-UTR-containing vectors did not reveal any reduction in luciferase activity (Figures 5C,D). Of note, luciferase activity in HEK293T cells that were transfected with the PsiCheck vector but did not receive any scrambled negative control was similar to the cells that were transfected with scrambled negative controls (Figure S4 in Supplementary Material).

To extend the analyses of miR181a and -b’s effects on Smad7 expression in lymphocytes, we transfected activated lymphocytes with miR-181a or -b mimics or negative control sequences. miR-181a overexpression significantly inhibited Smad7 expression (Figure 5E), whereas Socs3 and Tgfbr1 expression levels did not display a significant change (Figures 5F,G). Overall, these results implied that miR-181a regulated Smad7 but not Socs3 and Tgfbr1 expression.

### Smad7 Transcript Levels Are Negatively Correlated with miR-181a and -b Expression in CNS of EAE Mice and Activated Lymphocytes

**Figure 6:**
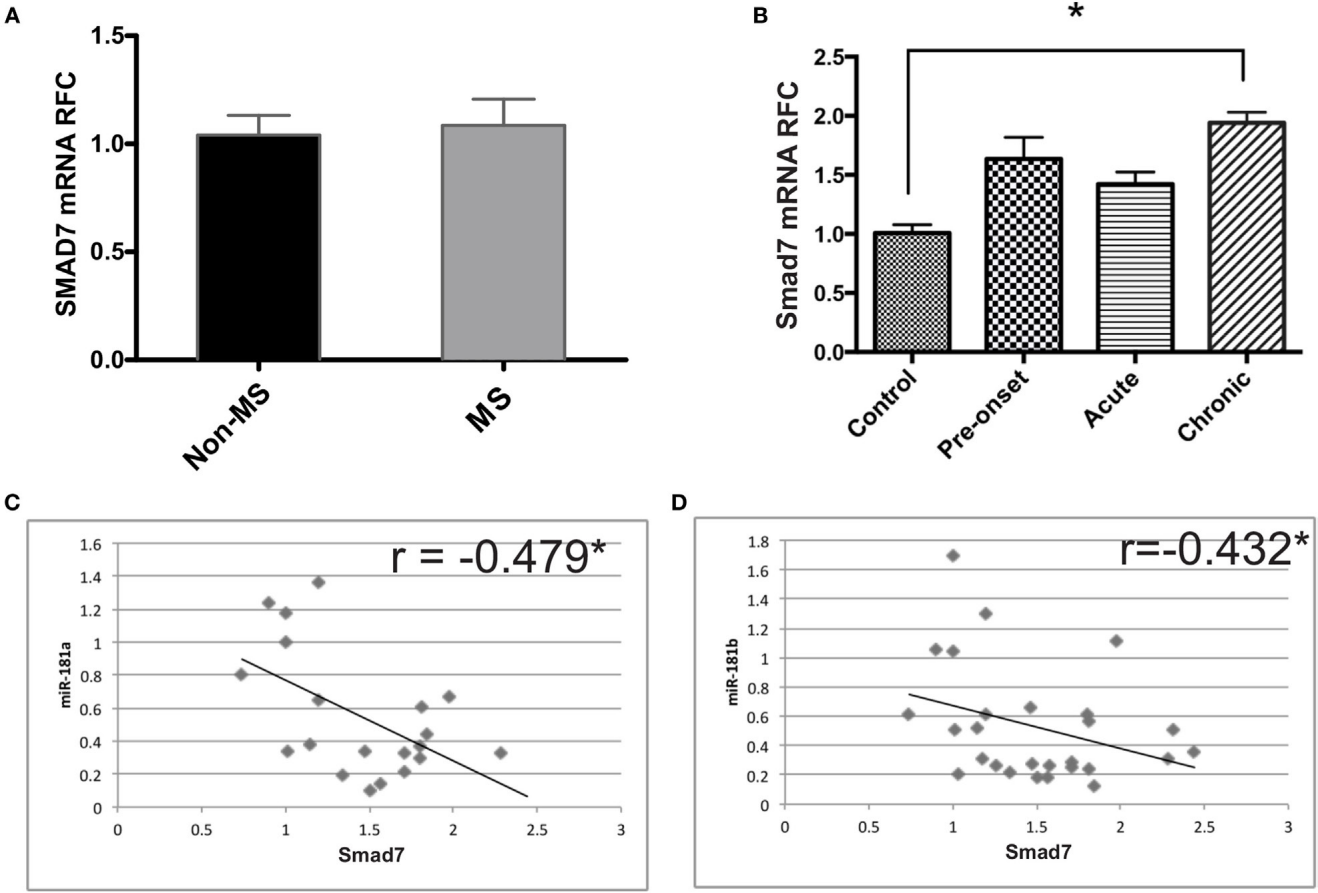
Expression of Smad7 genes is increased during the chronic phase of experimental autoimmune encephalomyelitis (EAE). Real-time PCR analysis shows the expression of SMAD7 in brain samples derived from multiple sclerosis (MS) patients **(A)**. Similar experiments were performed on spinal cord tissue from different phases of EAE **(B)**. Data are shown as mean ± SEM (*n* = 10 per group, ANOVA-Tukey *post hoc*; *p < 0.05). Correlation analysis was performed between Smad7 mRNA levels and miR-181a or miR-181b in acute and chronic phases of EAE **(C,D)** (Pearson correlation; *p < 0.05).

Given the decreased levels of miR-181a and -b in mice with EAE and the regulation of Smad7 expression by miR-181a and -b, we investigated Smad7 transcript levels in MS brain tissue as well as the lumbar spinal cord tissue of EAE mice. We did not observe any significant change in Smad7 transcripts in MS patients (Figure 6A). However, the data revealed significant rise of Smad7 mRNA levels in the chronic phase of EAE (Figure 6B). Correlation analyses revealed a significant inverse correlation between miR-181a or miR-181 b and Smad7 transcripts in EAE tissues (Figures 6C,D).

**Figure 7:**
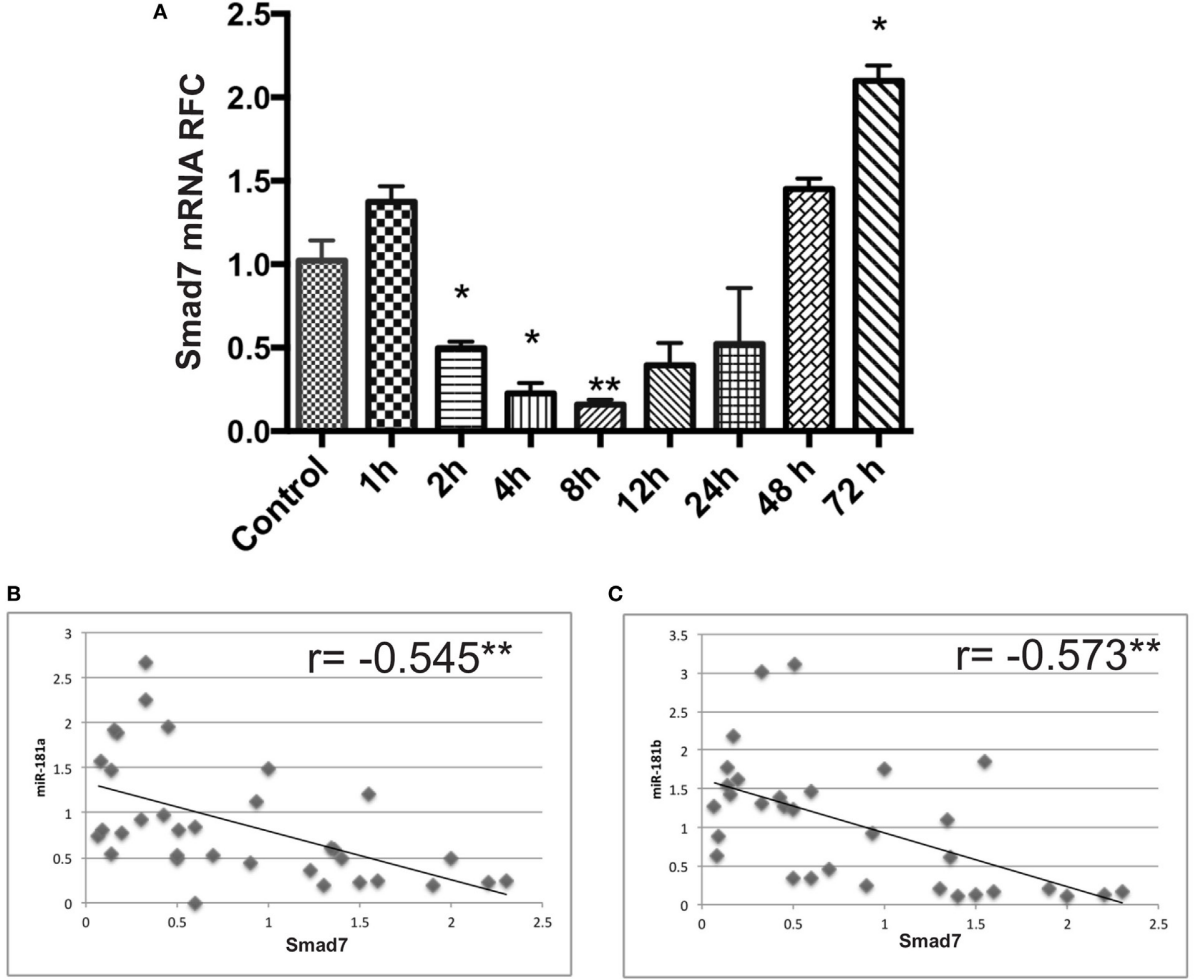
Smad7 transcript levels are dysregulated in anti-CD3/anti-CD28 activated lymphocytes at different time points. mRNA expression levels for Smad7 were measured by real-time RT PCR in activated T cells **(A)**. Correlation analysis between miR-181a/-b and Smad7 mRNA levels in activated lymphocytes are shown **(B,C)**. Data are shown as mean ± SEM (ANOVA-Tukey *post hoc* and Pearson correlation; *p < 0.05, **p < 0.01).

We also examined Smad7 levels in activated lymphocytes, which displayed an early suppression followed by a late upregulation at 48 and 72 h after activation (Figure 7A). This finding was associated with a significant negative correlation between Smad7 and miR-181a and -b expression levels (Figures 7B,C).

Overall these data showed that Smad7 expression was associated with alterations in miR-181 levels leading to molecular effects that influence T cell differentiation and macrophage activation in the context of autoimmune neuroinflammation.

## Discussion

Both adaptive and innate immune mechanisms have been implicated in development of neuroinflammation in MS. Among adaptive immune cells, myelin-reactive CD4^+^ and CD8^+^ T cells have been detected in blood and brain lesions of MS patients (1, 30). Studies comparing the proportions of T cell subsets have demonstrated a higher percentage of CD8^+^ T cells in MS lesions (30, 31). Nonetheless, data derived from studies on EAE animal model have given CD4^+^ T cells a more central role in disease process (32–34) with interferon-γ-producing Th1 and IL17-producing Th17 phenotypes being considered to be the key promoters of autoimmune neuroinflammation (1, 35–37). That said, the exact contribution of CD4^+^ versus CD8^+^ T cells in human disease is still a matter of debate (38). In addition to the lymphocytes, innate immune cells including infiltrating monocytes and resident microglia have been associated with MS pathogenesis. Similar to T cell differentiation, development of monocytoid cells toward M1 or M2 phenotypes has been considered important in the initiation and progression of inflammatory demyelination (39–41). Specific sets of cytokines and transcription factors that are responsible for T cell or macrophage differentiation have been identified; nonetheless, precise molecular mechanisms underlying perturbed T cell/macrophage polarization in MS or EAE have yet to be elucidated. In this study, we examined the role of miR-181a and -b in MS/EAE disease process. We provide evidence for miR-181a and -b suppression in the CNS in MS and EAE, as well as activated lymphocytes. We also show that increased levels of miR-181a and -b attenuate LPS-induced macrophage inflammatory responses and decrease the expression of M1-associated macrophage markers. Moreover, we demonstrate that miR-181a and -b could regulate the differentiation of T helper cells; miR-181a and -b both decrease differentiation toward a pathogenic Th1 phenotype and miR181a also increases generation of T regulatory cells. Our data indicate that miR-181a and -b might exert these effects by targeting Smad7.

The present findings of miR-181a and -b dysregulation in MS brains are in part consistent with previous miRNA studies in MS brains. Indeed, two miRNA expression profiling studies on tissues derived from MS patients have demonstrated differential expression of miR-181 family members in MS brain tissue (13, 14). A profiling study performed by Junker et al reported downregulation of miR-181 family members in MS lesions (13). However, in an miRNA profiling study performed by our group on NAWM from MS patients we detected increased levels of miR-181b in MS tissues (14). It should be noted that various factors might influence the miRNA content of diseased tissues. In inflamed tissues, the degree of inflammation and the level of leukocyte infiltration have been reported to affect the expression of various miRNAs (42–44). In our previous study, we performed miRNA profiling on tissues from seven MS cases as well as four control cases. As described in that report, MS cases were stratified into a “low-inflammation” group (4 cases) and a “high-inflammation” group (three cases), based on the levels of inflammatory transcripts. Tissues exhibiting lower levels of inflammatory transcripts (i.e., less leukocyte infiltration and microglial cell activation) were examined further to detect neural cell-related miRNAs with a role in neurosteroid synthesis. We believe that miR-181b increase reported in our previous study was mostly reflective of miRNA content of neural cells (e.g., astrocytes and neurons). Of interest, the “high-inflammation” group of MS patients revealed an miRNA expression pattern which was distinct from both controls and the “low-inflammation” group (unpublished data). In addition to the level of inflammation, another potential factor in determining the miRNA content might be the stage of T cell activation. Our current data show that miR181a is induced in the spinal cords of EAE mice right before the onset of symptoms and then it shows a decrease in the acute and chronic phases (Figure 1I). Moreover, miR181a and -b both show a degree of upregulation in activated lymphocytes before their levels get suppressed (Figures 2D,E), a phenomenon which might be indicative of the presence of a complex miRNA regulatory system in leukocytes. So it seems that the stage of T cell activation and the dynamics of the lesion formation are other determinants of miR181a and -b levels.

Our experiments on monocytoid cells showed that miR-181a and -b diminished proinflammatory cytokine production and differentiation toward the M1 phenotype. These observations are consistent with previous studies, which have demonstrated anti-inflammatory roles for miR-181a (45, 46). In a study on monocytes and macrophages, Xie et al. (45) showed anti-inflammatory effects for miR-181a by targeting IL1α in mice and human monocytes and macrophages (45). miR-181a has also been shown to attenuate the ox-LDL-stimulated immune inflammatory response in dendritic cells by targeting c-fos (47). Studies on mouse astrocytes have also indicated a regulatory role for miR-181 family members in these cells (46). In addition to monocytoid cells, we observed effects of miR-181a and -b on T helper cell phenotypic development, whereby both miR-181a and -b decreased Th1 cells differentiation and miR-181a promoted Treg cell generation. The impact of miR-181a and -b on lymphopoiesis and T cell development has been elucidated by previous studies (10, 19, 48–50). miR-181-deficient mice show severe defects in development of B, T, NK and NKT cells (49). Li et al. have shown that enhanced expression of miR-181a and -b in different stages of T cell development promotes positive and negative selection through enhancing TCR sensitivity and signaling strength (19). Schaffert et al. have reported that mir-181a-1/b-1 deletion in mice leads to the selection of more autoreactive T cells and higher reactivity of peripheral T cells to self-antigens. Interestingly, in their study mir-181a-1/b-1 deletion augmented Th1 differentiation *in vitro* and also caused an increase in Th1 and Th17 differentiation in the spleens of EAE mice while there was no difference in Treg differentiation and function (51). These findings are in agreement with our results which also point to the inhibitory effect of miR-181a and -b on Th1 polarization. However, in contrast to the effect on Th1 differentiation, mir-181a-1/b-1-deficient mice showed a delayed and attenuated EAE phenotype which was a consequence of inhibition of T cell migration into the CNS. Similarly, inhibition of miR-181a by an antagomir delayed and dampened EAE disease (51). So it seems that while miR-181a and -b diminish differentiation of T cells toward the pathogenic Th1/Th17 phenotypes, they are also required for efficient infiltration of CNS by myelin-reactive T cells. These two opposing effects on the pathogenesis need to be considered when manipulating miR-181a and -b levels *in vivo*.

miR-181a and -b have multiple predicted mRNA targets with known roles in immune response. These targets include signaling molecules belonging to the Smad, STAT, TGF-b receptor and SOCS signaling pathways as well as cytokines including IL-2, IL-6, IL-7, and IFNγ. In this study, using broad conservation of miRNA binding sites across species and known effects on T cell differentiation as selection criteria, we chose to study three transcripts, i.e., Socs3, Tgfbr1, and Smad7, as potential targets of miR-181a and -b. SOCS proteins negatively regulate Janus kinase/signal transducer and activator of transcription pathways (Jak/STAT). SOCS3 is an inhibitor of STAT3 that is essential for induction of the orphan nuclear receptor RORt, Th17 polarization and EAE development (52–54). Hence, SOCS3 is assumed to be a negative regulator of Th17 differentiation (28) Transforming growth factor (TGF)-β1, a master regulator of immune responses (55, 56), inhibits the Th1 and Th2 cells differentiation of by suppressing T-bet and GATA-3, respectively (57), whereas it promotes regulatory T cell generation by upregulating Foxp3 (58). Loss of TGF-β signaling in T cells results in the T cell activation, expansion of autoreactive T cells and decreased peripheral numbers of regulatory T cells (56, 59). Finally, the Smad family of proteins mediate TGFβ signaling (60). Smad7, an inhibitor of TGF-β signaling (61, 62), prevents the phosphorylation of other Smad proteins (63). Smad7 positively regulates *in vitro* differentiation of Th1 cells. Previous studies have shown that Smad7 in T cells promotes Th1 responses in MS and EAE (27). Furthermore, enhanced infiltration of regulatory T cells to the CNS of Smad7 deficient mice has been observed (27). In the present study, molecular assays using 3′-UTR-luciferase vectors showed suppression of Smad7 by miR-181a and -b, whereas the results revealed no significant interaction between miR-181a/-b and Socs3 or Tgfbr1 mRNAs. Consistently, Smad7 transcript levels were significantly increased in the spinal cords of EAE mice in the chronic phase of disease and an overall negative correlation between Smad7 transcript levels with miR-181a and -b was detected. However, SMAD7 transcript levels did not show an increase in MS brain tissue. Upregulation of Smad7 protein in CD4^+^ T cells infiltrating the brain as well as in EAE spinal cords has been reported before (27, 64). Considering that miRNAs might affect the expression of genes at the translational level the possibility of regulating Smad7 protein levels by miR-181a and -b remains. Smad7 transcript levels in activated lymphocytes showed an initial decrease at earlier time points before it was upregulated at 48- and 72-h time points. This upregulation at later time points was consistent with the suppression of miR-181a and -b in activated lymphocyte at the same time points and an overall negative correlation was observed between Smad7 and miR-181a and -b levels in these cells.

Altogether, results of our expression analyses in CNS tissue, miRNA overexpression studies in macrophages and lymphocytes and mRNA target identification and expression analyses suggest that miR-181a and -b modulate immune responses through their anti-inflammatory effects on T cell differentiation and also macrophages, phenomena which might be of therapeutic value in autoimmune neuroinflammation as well other inflammatory disorders associated with mononuclear cell activation.

## Ethics Statement

The use of autopsied brain tissues were approved under the protocol number 2291 by the University of Alberta Human Research Ethics Board (Biomedical), and written informed consent documents were signed for all samples. All animal experiments were performed in accordance with guidelines from Animal Care Committee of Tehran University of Medical Sciences.

## Author Contributions

SG performed the experiments, analyzed the results, and wrote the manuscript. FT and FM helped with the experiments and data analysis. WFC performed gene expression experiments on human tissues. MV and CP supervised the research process, which included human, animal, and *in vitro* studies. FN developed the hypothesis, designed the project, and supervised the research. CP and FN edited the final manuscript.

## Conflict of Interest Statement

The authors declare that the research was conducted in the absence of any commercial or financial relationships that could be construed as a potential conflict of interest.

## Supplementary Material

Table S1 Disease phenotype and age/sex statistics of multiple sclerosis (MS) patients and non-MS controls.

Table S2 Primer sequences for real-time RT-PCR.

Figure S1 miR-181a and miR-181b expression levels after transfection with miR-181a and miR-181b mimics.

Figure S2 Activation of T cells following anti-CD3/CD28 treatment was confirmed by checking the proliferation of CD3 positive cells using CFSE staining **(A)** and also measuring their IL2 **(B)** and interferon gamma **(C)** expression by real-time PCR.

Figure S3 The expression of inflammatory cytokines and M1/M2 markers in resting macrophages (without LPS treatment) following miR-181a and -b mimic transfection.

Figure S4 The RLU for vector-transfected HEK cells in untransfected cells (mock) and scrambled sequence-transfected cells. Both groups of cells have been transfected with luciferase vector.
