# Supplementary material for: MicroRNA-181 Variants Regulate T Cell Phenotype in the Context of Autoimmune Neuroinflammation
Source: Front Immunol. 2017 Jul 19;8:758. doi: 10.3389/fimmu.2017.00758 (PMC5515858; doi:10.3389/fimmu.2017.00758)
Supplement: Table S1 — Disease phenotype and age/sex statistics of multiple sclerosis (MS) patients and non-MS controls. [file Table_1.PDF]

	**MS (n=10)**	**nonMS (n=10)**	***p* value***
**Mean age (range, yrs.)**	58 (36-74)	62 (47-74)	NS
**M:F**	6:4	6:4	NS
**Disease phenotype**	PP-MS[1] (n=2)<br>SP-MS[3] (n=8)	Stroke (n=1),<br>ALS (n=2),<br>nonCNS disease (cancer, sepsis, myocardial infarction, n=7)	
**Disease Duration (range, yrs.)**	5-20	1-5	
**Therapy**		(cancer chemotherapy, n=3; antibiotics, n=1)	
**EDSS**	7.0-9.5	N/A	
