# Supplementary material for: MicroRNA-181 Variants Regulate T Cell Phenotype in the Context of Autoimmune Neuroinflammation
Source: Front Immunol. 2017 Jul 19;8:758. doi: 10.3389/fimmu.2017.00758 (PMC5515858; doi:10.3389/fimmu.2017.00758)
Supplement: Table S2 — Primer sequences for real-time RT-PCR. [file Table_2.PDF]

**Official Gene Symbol(Refseq)**	**Primers**
mmu-TNF-a	F:CCAGTGTGGGAAGCTGTCTT<br>R:AAGCAAAAGAGGAGGCAACA
mmu-IL1a	F:GCACCTTACACCTACCAGAGT<br>R:AAACTTCTGCCTGACGAGCTT
mmu-IL1b	F:TGAAGAAGAGCCCATCCTCTG<br>R:GGAGCCTGTAGTGCAGTTGT
mmu-IL6	F:TCCAGTTGCCTTCTTGGGAC<br>R:GTGTAATTAAGCCTCCGACTTG
mmu-CD3e	F:ATGCGGTGGAACACTTTCTGG<br>R:GCACGTCAACTCTACACTGGT
mmu-GFAP	F:GGGGCAAAAGCACCAAAGAAG<br>R:GGGACAACTTGTATTGTGAGCC
mmu-F4/80	F:TTGTACGTGCAACTCAGGACT<br>R:GATCCCAGAGTGTTGATGCAA
mmu-IL2	F:TGAGCAGGATGGAGAATTACAGG<br>R:GTCCAAGTTCATCTTCTAGGCAC
mmu-actin	F:ATGCTCCCCGGGCTGTAT<br>R:CATAGGAGTCCTTCTGACCCATTC
mmu-IFNg	F: ATGAACGCTACACACTGCATC<br>R: CCATCCTTTTGCCAGTTCCTC
mmu-GAPDH	F: TGTGTCCGTCGTGGATCTGA<br>R: TTGCTGTTGAAGTCGCAGGAG
mmu-iNOS	F:GGCAGCCTGTGAGACCTTTG<br>R:GCATTGGAAGTGAAGCGTTTC
mmu-Arg1	F:AGCACTGAGGAAAGCTGGTC<br>R:CAGACCGTGGGTTCTTCACA
mmu-Mrc1	F:GTGGAGTGATGGAACCCCAG<br>R:CTGTCCGCCCAGTATCCATC
mmu-TGFbR1	F:GGCGAAGGCATTACAGTGTT<br>R:AAGGCCAGCTGACTGCTTTT
	F:TGTCGGAAGACTGTCAACGG

mmu-SOCS3	R:GAAGAAGCCAATCTGCCCCT
mmu-Smad7	F:GGCCGGATCTCAGGCATTC<br>R:TTGGGTATCTGGAGTAAGGAGG
