# Supplementary material for: MicroRNA-181 Variants Regulate T Cell Phenotype in the Context of Autoimmune Neuroinflammation
Source: Front Immunol. 2017 Jul 19;8:758. doi: 10.3389/fimmu.2017.00758 (PMC5515858; doi:10.3389/fimmu.2017.00758)
Supplement: Figure S1 — miR-181a and miR-181b expression levels after transfection with miR-181a and miR-181b mimics. [file Presentation_1.pptx]

## Slide 1
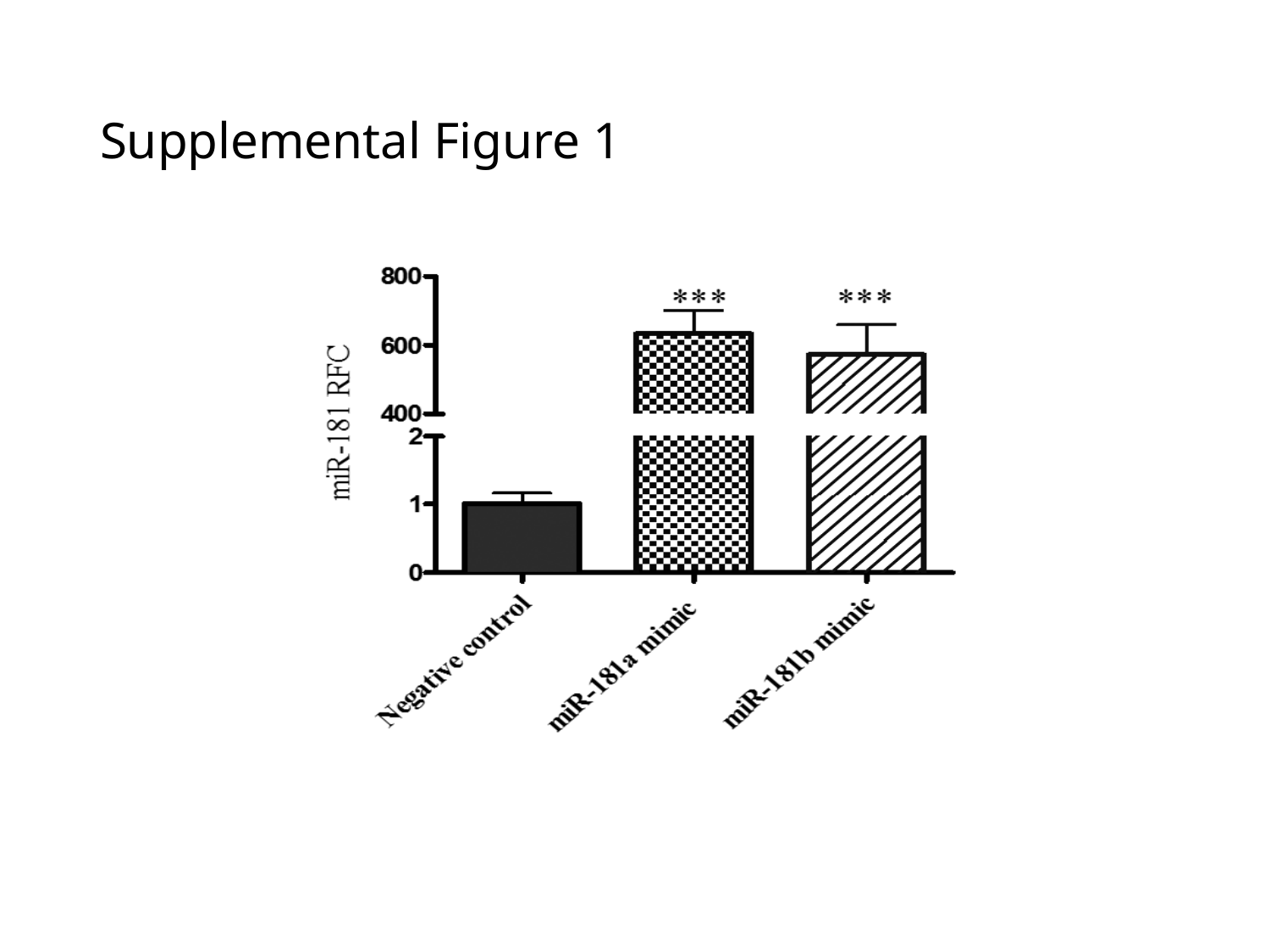

# Supplemental Figure 1

## Slide 2
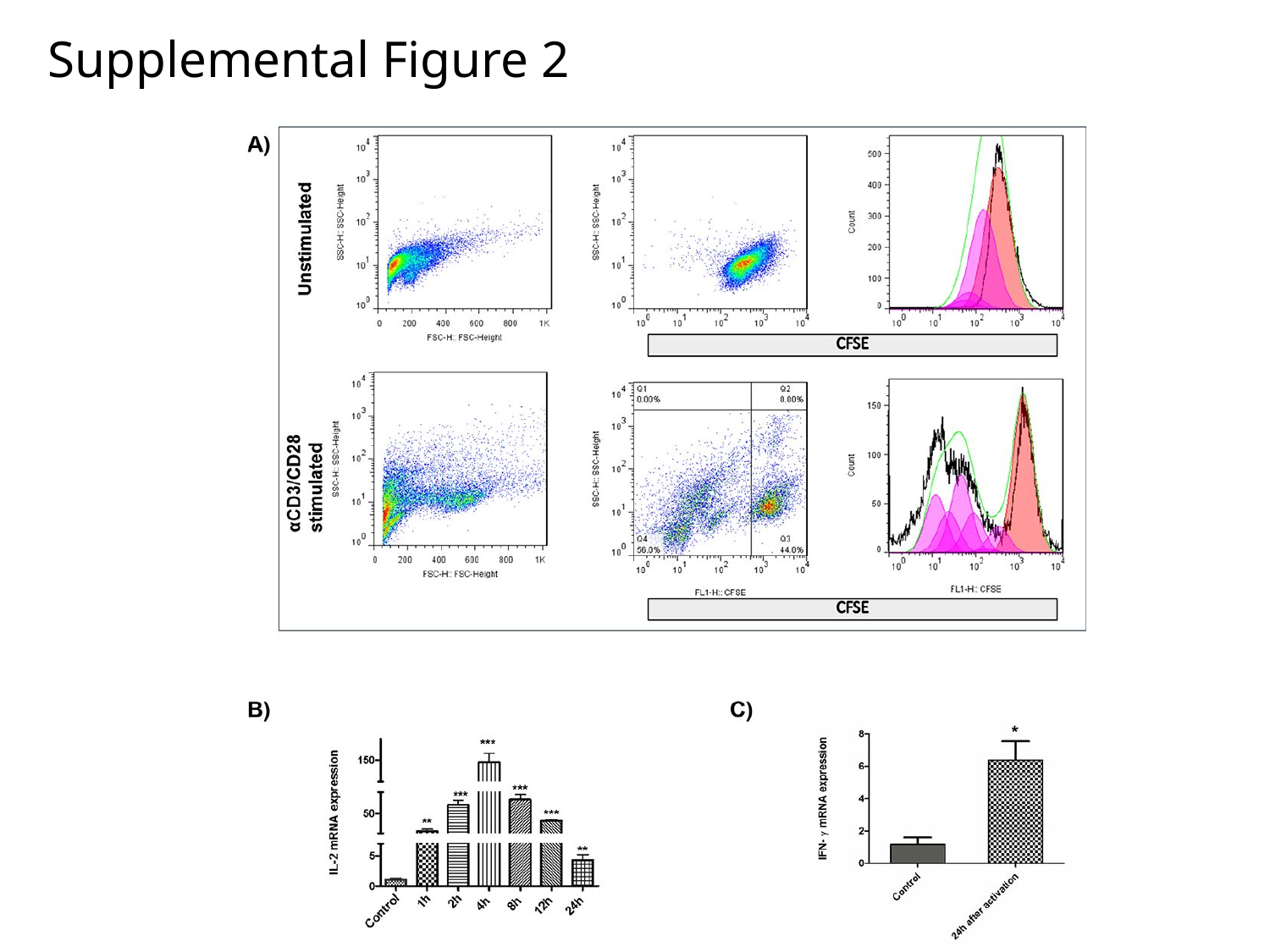

# Supplemental Figure 2

## Slide 3
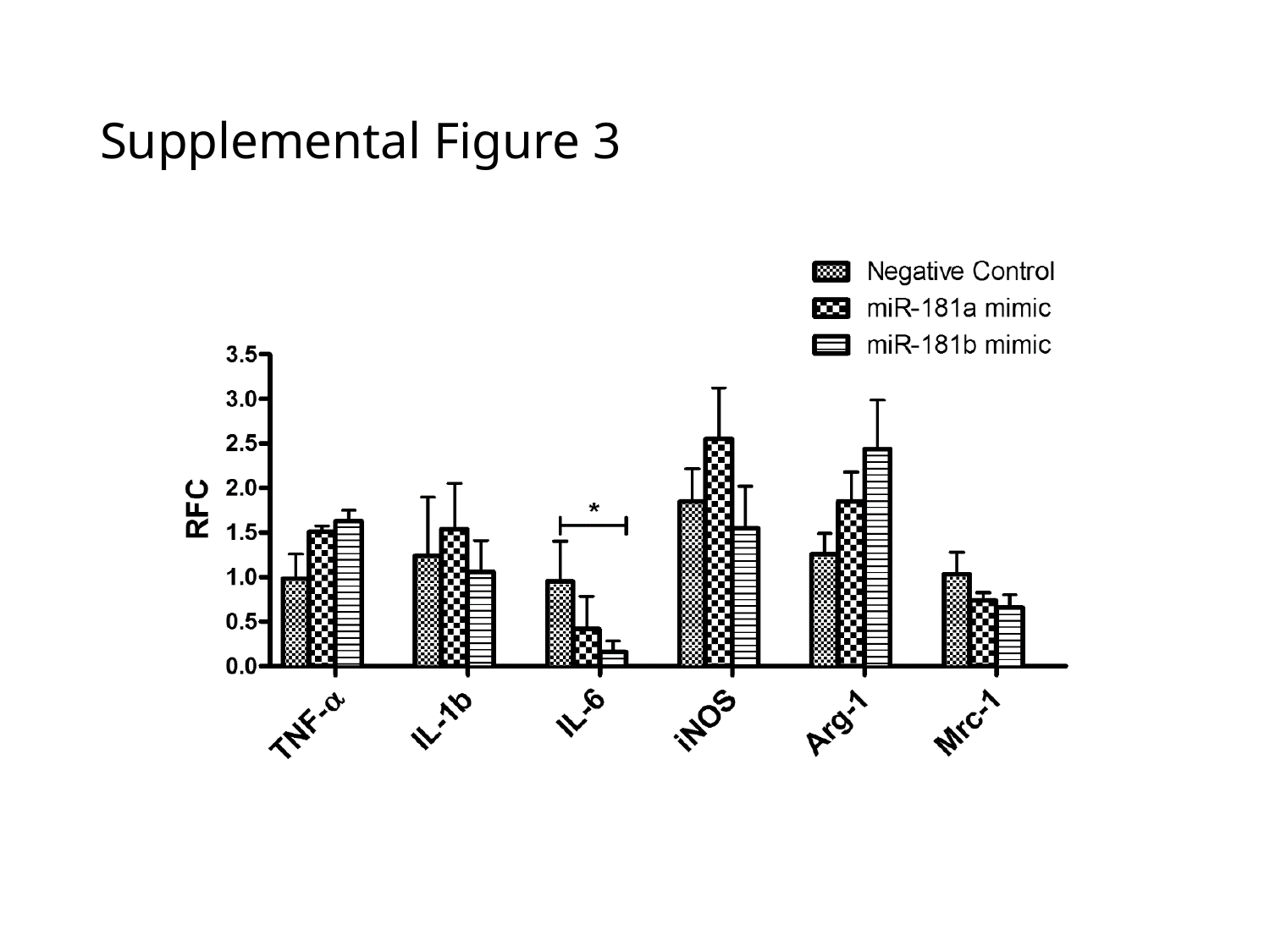

# Supplemental Figure 3

## Slide 4
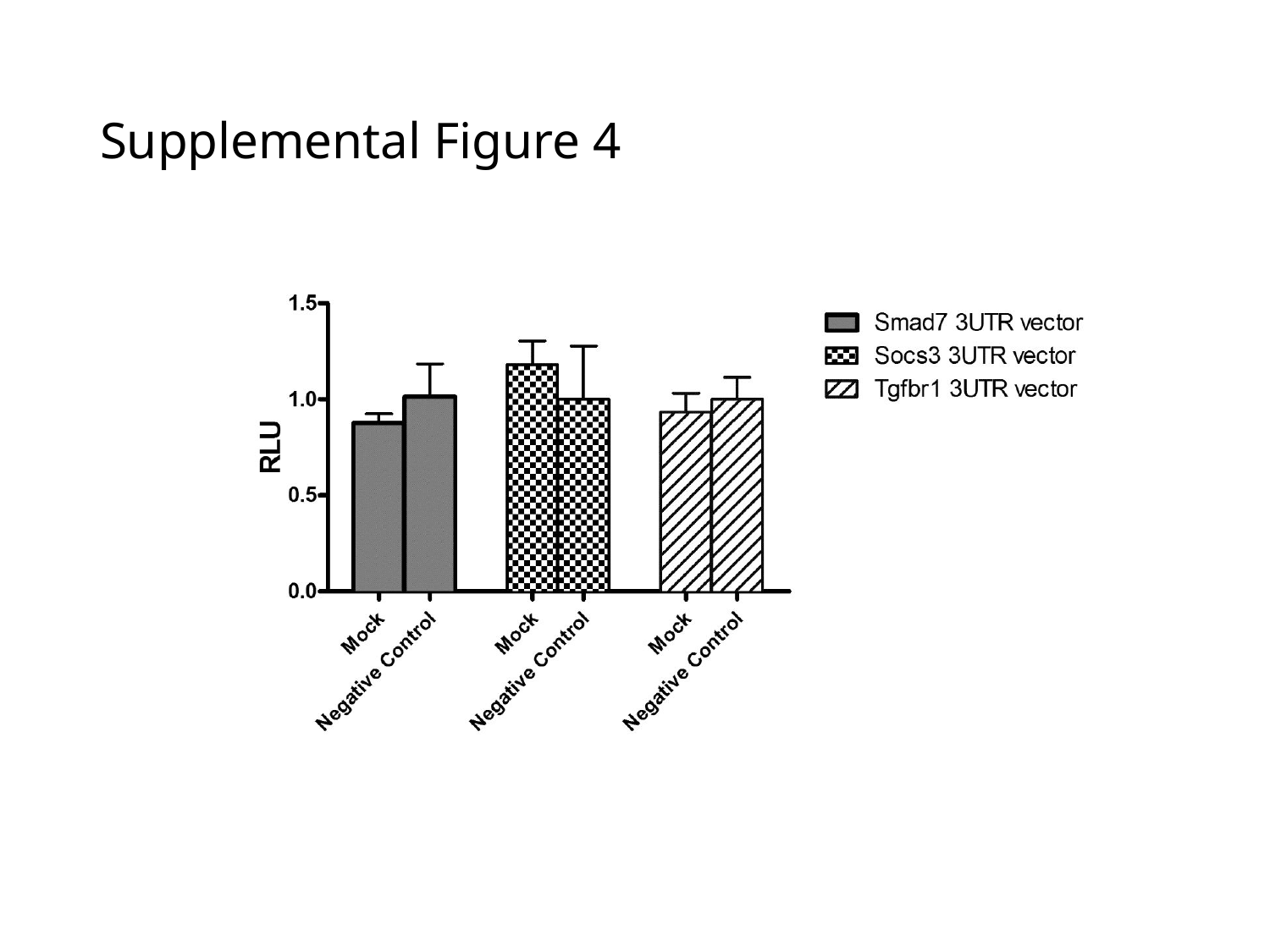

# Supplemental Figure 4
